# Effects of a senior-friendly diet on health-related quality of life and quality-adjusted life expectancy among older adults in South Korea: a fixed-effects and difference-in-differences analysis

**DOI:** 10.3389/fpubh.2026.1846215

**Published:** 2026-06-12

**Authors:** Hee-Sook Lim, Hansol Park, Min-Jung Bae, MoonYeon Baek, Kyeong-Hee Choi, Namhoon Kim, Sanghyo Kim

**Affiliations:** 1 Department of Gerontology, AgeTech-Service Convergence Major, Graduate School of East-West Medical Science, Kyung Hee University, Dongdaemun, Republic of Korea; 2 The Food Industry Promotion Agency, Iksan-si, Republic of Korea; 3 Department of Food and Resource Economics, Pusan National University, Geumjeong-gu, Republic of Korea; 4 Korea Rural Economic Institute, Naju, Republic of Korea

**Keywords:** difference-in-differences, fixed-effects, QALY, quality of life, senior-friendly food, utility weight

## Abstract

**Introduction:**

Rapid population aging presents significant nutritional challenges for older adults, as physiological declines often lead to malnutrition and reduced quality of life. While senior-friendly diets have been developed to accommodate these needs, their broader economic and welfare impacts remain under-researched.

**Methods:**

To address this gap, a randomized two-period crossover intervention study was conducted with community-dwelling older adults at a welfare center in South Korea. Participants received either a senior-friendly diet or a standard diet for ten weeks before switching conditions after a one-week washout period. Using individual fixed-effects models as the primary analysis and difference-in-differences models as a robustness check, changes in utility weights and quality-adjusted life expectancy were analyzed.

**Results:**

The results demonstrated that consuming the senior-friendly diet led to statistically significant improvements in both utility weights and quality-adjusted life expectancy. These overall gains were primarily driven by significant enhancements in physical functioning, specifically in mobility, self-care, usual activities, and the reduction of pain and discomfort.

**Discussion:**

These findings suggest that senior-friendly dietary interventions may improve physical health and quality-adjusted life expectancy among older adults in community-based welfare settings, though further research with larger and more diverse samples is needed to assess the generalizability of these effects.

## 1 Introduction

Population aging has emerged as one of the most significant demographic transformations of the 21st-century (1). South Korea, in particular, is experiencing an exceptionally rapid demographic transition toward an aging society (1). In 1970, individuals aged 65 years and older accounted for only 3.1% of the total population. However, by 2022, this proportion had increased dramatically to 17.5% (2). Demographic projections suggest that this trend will continue to accelerate, with the older adult population expected to reach 34.4% of the total population by 2040 and 46.4% by 2070 (2). This pace of population aging is considerably faster than that observed in many other developed countries (1, 2).

The rapid growth of the older adult population presents substantial challenges for social welfare systems, public health policy, and food systems. As populations age, maintaining the health and well-being of older adults becomes increasingly important for sustainable social development. Among the many determinants of healthy aging, adequate nutrition plays a particularly critical role. Proper dietary intake contributes to maintaining physical health, preventing chronic diseases, and sustaining functional independence among older adults. Grunert et al. (3) and Shin et al. (4) emphasized the critical role of adequate nutrition in supporting healthy aging.

However, despite the growing importance of older adult nutrition, government policies and societal responses in South Korea have not evolved at the same pace as the demographic transition. As the population ages, the demand for nutritional care has increased significantly, yet ensuring adequate nutritional intake among older adults remains a persistent challenge. Many older adults face difficulties related to food intake due to physiological and functional limitations. For example, swallowing disorders, chewing difficulties, and cognitive impairments can reduce food intake and hinder the ability to prepare balanced meals. Doan et al. (5) and Namasivayam et al. (6) documented these functional limitations as major barriers to adequate nutrition in older adults.

These challenges are particularly pronounced among older adults individuals in poor health or those living alone or in institutional settings. Age-related impairments in chewing and swallowing functions can lead to reduced dietary diversity and insufficient intake of essential nutrients. As a result, older adults are more vulnerable to malnutrition, frailty, and chronic diseases associated with inadequate nutrition. Previous studies have consistently reported this increased vulnerability (7–9).

In response to these concerns, increasing attention has been directed toward the development of senior-friendly foods. In South Korea, senior-friendly foods refer to food products that are modified in either their physical composition or nutritional content in order to improve ease of intake among older adult consumers. These foods typically involve adjustments in texture, hardness, moisture, or nutrient composition to accommodate the physiological needs of aging individuals (10). The market for senior-friendly foods has recently begun to attract considerable interest from both policymakers and industry stakeholders. As awareness of older adult nutrition grows, demand for foods designed specifically for older consumers is expected to increase significantly in the coming years. Senior-friendly foods are considered an important solution for addressing nutritional challenges associated with aging, as they enable older adults to consume essential calories and nutrients more easily.

From a public health perspective, these custom-designed food products have the potential to play a crucial role in improving the nutritional status and physical health of older adult populations. By improving accessibility to nutritionally balanced and easily consumable foods, senior-friendly diets may contribute to reducing malnutrition and improving functional health among older adults (4, 11). In addition to health benefits, the development of senior-friendly food products also presents important policy and economic implications. As the market expands, governments may introduce policies aimed at promoting the production and certification of high-quality senior-friendly foods. Such policies could support the development of a specialized food industry while simultaneously improving nutritional outcomes for older adult populations.

Despite the growing interest in senior-friendly foods, several important research gaps remain in existing literature. Most previous studies have focused primarily on technological innovations and nutritional formulations in older adult-friendly food development, particularly the design of texture-modified foods aimed at addressing chewing and swallowing difficulties among older adults (12, 13). While these studies provide valuable insights into food product development, relatively little attention has been paid to the broader welfare implications of consuming senior-friendly foods. In particular, empirical evidence examining how senior-friendly diets influence the quality of life of older adults remains limited. Quality of life is widely recognized as a multidimensional concept encompassing physical health, psychological well-being, and social participation (14, 15).

Furthermore, there is a lack of economic analyses that quantify the welfare value of senior-friendly diets. Understanding the economic implications of dietary interventions is important for designing effective policies that promote healthy aging and sustainable food systems. Without such evidence, policymakers face difficulties in assessing the social value of investing in senior-friendly food programs. In health economics, Quality-Adjusted Life Years (QALYs) are widely used to evaluate the welfare impacts of health interventions by integrating both longevity and quality-of-life outcomes (14).

Against this background, the purpose of this study is to empirically evaluate the economic and welfare implications of introducing senior-friendly foods. Using data from a randomized crossover intervention study, this study investigates how exposure to senior-friendly diets affects the quality of life of older adults. In addition, this study quantifies the welfare benefits of senior-friendly foods by incorporating utility weights and estimating their effects in terms of Quality-Adjusted Life Years (QALYs). By linking nutrition, welfare policy, and economic evaluation, this study contributes to literature in several ways. First, it provides empirical evidence on the relationship between senior-friendly diets and quality of life among older adult populations. Second, it introduces an economic evaluation framework based on QALY measures to quantify the welfare benefits of dietary interventions. Finally, the findings offer policy-relevant insights into how senior-friendly food systems can contribute to improving the well-being of older adult populations in rapidly aging societies.

## 2 Literature review

### 2.1 Nutritional challenges and dietary risks among the older adults

Aging is associated with significant physiological and metabolic changes that affect dietary behavior and nutritional status. Declining appetite, reduced digestive efficiency, and decreased sensory perception often lead to reduced food intake among older adults. As a result, older adult populations are particularly vulnerable to malnutrition and nutritional imbalance (4, 8). Consistent with this concern, Lim et al. (28) revised the Korean NQ-E as a brief tool for assessing dietary quality and dietary behavior across balance, moderation, and practice domains among older adults.

One of the most critical challenges in older adult nutrition is the deterioration of oral and swallowing functions. Mastication difficulties and dysphagia are common conditions among older adults and are widely recognized as major barriers to adequate dietary intake. Studies estimate that swallowing disorders affect a substantial proportion of older adults, particularly those living in long-term care facilities or suffering from chronic diseases (5, 6). These conditions can significantly reduce food consumption and limit dietary diversity. Bayram et al. (27) similarly found that older adults with dysphagia had greater nutritional risk, lower hand-grip strength, and inadequate energy and micronutrient intake compared with nondysphagic controls.

Chewing impairment is another important factor affecting dietary quality among older adults. Individuals with reduced chewing ability often avoid harder foods such as fresh vegetables, fruits, and meat, which are important sources of essential nutrients. Consequently, older adults with mastication difficulties tend to have lower intake of protein, fiber, and micronutrients, increasing their vulnerability to malnutrition and chronic diseases (7, 9).

Poor oral health also contributes to nutritional vulnerability among older populations. Tooth loss, periodontal disease, and reduced salivary function can significantly impair masticatory efficiency and alter food preferences. Previous research has shown strong associations between oral health status, dietary intake, and frailty among older adults (7, 16).

The nutritional consequences of these intake difficulties extend beyond dietary imbalance. Inadequate nutrient intake has been linked to increased risks of sarcopenia, frailty, cardiovascular disease, and metabolic disorders in older adult populations (8). As such, improving dietary intake among older adults has become an important public health priority in rapidly aging societies.

### 2.2 Development and characteristics of senior-friendly foods

To address the nutritional challenges faced by older adult populations, researchers and policymakers have increasingly emphasized the development of senior-friendly foods. Senior-friendly foods are designed to accommodate the physiological limitations associated with aging while ensuring adequate nutritional intake. Various product-development approaches, including texture modification and sensory evaluation of senior-friendly foods, have been explored to improve food accessibility and acceptability for aging populations (13, 17).

In addition to texture modification, senior-friendly diets often incorporate protein enrichment and micronutrient fortification in order to prevent age-related muscle loss and nutritional deficiencies. Increased intake of high-quality protein has been shown to improve muscle mass, physical function, and metabolic health among older adults (4).

Technological innovations in food processing have further advanced the development of senior-friendly foods. Emerging technologies such as high-pressure processing, modified atmosphere cooking, and food 3D printing enable the production of customized foods that meet both nutritional and sensory requirements of older adult consumers (12, 13). These innovations have expanded the possibilities for producing nutritionally balanced and easy-to-consume foods tailored to individual needs.

Consumer acceptance is also an important consideration in the development of senior-friendly foods. Previous research indicates that older adult consumers generally prefer foods that preserve familiar sensory characteristics while incorporating texture modifications that facilitate easier chewing and swallowing (3, 17).

### 2.3 Institutional food services and older adult nutrition

Institutional food services play a crucial role in delivering nutritional support to older adult populations, particularly those living in welfare facilities or long-term care institutions. These facilities often serve older adults who face difficulties preparing meals independently due to physical limitations or social isolation.

In South Korea, welfare facilities have increasingly adopted structured food service systems designed to improve nutritional management for older adult residents. One common approach is the implementation of centralized kitchen systems, which allow institutions to standardize meal preparation and maintain consistent nutritional quality across facilities (18).

Centralized food service systems offer several advantages, including improved operational efficiency, standardized portion sizes, and improved quality control in food preparation. However, large-scale production and extended holding times may pose challenges for maintaining nutrient quality if appropriate management practices are not implemented (19, 20).

To address these challenges, specialized nutrition management organizations such as the Center for Social Welfare Foodservice Management (CSWFM) provide professional support for welfare facilities. These institutions assist with menu planning, nutrition education, and food safety management, thereby improving the overall quality of institutional food services (19).

In addition to institutional meal services, home-delivered meal programs and community-based nutrition interventions have also been implemented to improve dietary outcomes among vulnerable older adult populations. These programs have been shown to improve dietary intake, reduce malnutrition, and enhance subjective health status among older adults living alone or facing economic hardship (21, 22).

### 2.4 Effects of senior-friendly diets on health and quality of life

A growing body of research has examined the health outcomes associated with senior-friendly dietary interventions. Empirical studies suggest that improving dietary intake among older adult populations can significantly reduce malnutrition and frailty while improving physical functioning.

For example, Shin et al. (4) found that senior-friendly dietary interventions increased energy and protein intake among older adults and improved indicators of physical function, including muscle mass and gait speed. Similarly, protein-enriched diets have been shown to slow age-related decline in nutritional status and improve physical health outcomes among older adult residents in long-term care facilities (11).

Beyond physical health outcomes, nutrition interventions may also influence psychological well-being and social engagement among older adult populations. Meal programs that improve dietary quality have been shown to reduce depression and improve life satisfaction among older adults, particularly those who experience social isolation (15, 23).

Social aspects of food consumption also play an important role in older adults well-being. Communal meal programs and social dining activities can strengthen social networks and enhance subjective quality of life among older adults (3). As such, dietary interventions may contribute not only to improved physical health but also to broader improvements in life satisfaction and social well-being.

### 2.5 Economic evaluation and the role of quality of life measures

While the health benefits of nutritional interventions have been widely studied, relatively few studies have examined the economic value of dietary interventions for older adult populations. From a policy perspective, understanding the economic benefits of nutrition programs is essential for evaluating the cost-effectiveness of public health interventions.

Quality-Adjusted Life Years (QALYs) have become one of the most widely used measures for evaluating health outcomes in economic evaluations (14, 24). The QALY framework combines both the quantity and quality of life into a single metric, allowing researchers to assess the welfare benefits of medical or public health interventions.

Previous studies have applied QALY-based approaches to evaluate a variety of health interventions, including pharmaceutical treatments, disease prevention programs, and long-term care services. However, the application of QALY-based evaluation to dietary interventions for older adult populations remains relatively limited.

Given that nutrition affects both physical health and subjective well-being, dietary interventions are particularly suitable for evaluation using QALY frameworks. Improvements in nutritional status may lead not only to reduced disease risk but also to enhanced life satisfaction and functional independence among older adults.

Despite this potential, empirical studies that quantify the welfare benefits of senior-friendly diets using QALY-based approaches remain scarce. As a result, policymakers currently lack sufficient evidence to evaluate the broader social value of investing in senior-friendly food systems.

Building on the existing literature, this study aims to fill an important gap by empirically evaluating the welfare effects of senior-friendly diets using a QALY-based framework. Specifically, this study investigates how exposure to senior-friendly diets influences the quality of life of older adults living in welfare facilities in South Korea. By incorporating utility weights and estimating changes in Quality-Adjusted Life Years, the analysis provides a quantitative assessment of the welfare benefits associated with dietary interventions designed for older adult populations.

## 3 Method

### 3.1 Experimental design and participants

We conducted a community-based randomized crossover intervention study among older adults receiving care at a welfare center. The participants were community-dwelling older adults who attended Baekhwa Senior Welfare Center and generally exhibited mild or no severe limitations in physical or cognitive functioning. In this study, older adults were defined as individuals aged 65 years and older, reflecting the standard threshold used in aging-related research and policy discussions on global demographic change.

**Figure 1:**
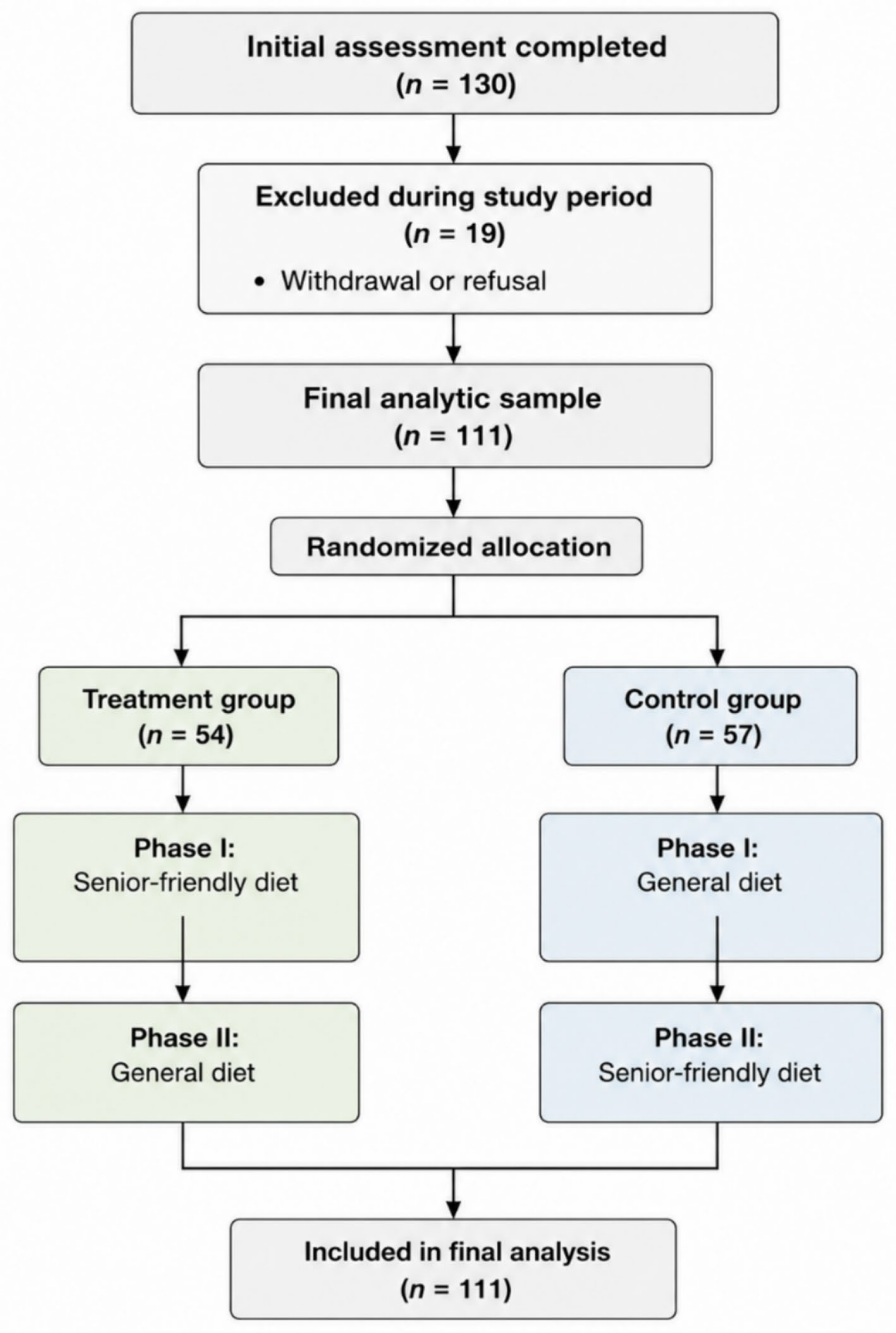
CONSORT-style participant flow diagram of the randomized crossover intervention study.

A total of 111 participants were enrolled in the study. Participants were randomly assigned to either the treatment group (*n* = 54) or the control group (*n* = 57) at baseline. Pre- and post-tests were conducted at each measurement point. Of 130 individuals who completed the initial assessment, 19 were excluded due to withdrawal or refusal during the study period, generating a final analytic sample of 111 participants. No missing data were observed for any of the variables used in the analysis among the final sample (see Figure 1).

The study followed a randomized two-period crossover intervention design with a washing-out interval between phases. Each intervention phase lasted 10 weeks, and the washing-out period lasted 1 week. This structure allowed participants to receive both the general diet and the senior-friendly diet at different points in time, improving statistical efficiency while controlling for individual-level heterogeneity.

In Phase I, participants assigned to the treatment group received the senior-friendly diet, while those assigned to the control group received the general diet. After the 10-week intervention and the 1-week washing-out period, the groups switched conditions in Phase II. Participants who had previously received the senior-friendly diet were provided with the general diet, and those who had previously received the general diet were provided the senior-friendly diet. The washing-out period was introduced to mitigate potential residual effects from the first intervention period.

All participants attended the welfare center regularly and were provided with daily lunch meals. The control condition consisted of the standard lunch menu typically provided at the center, which was designed to meet the general nutritional needs of older adults. The treatment conditions consisted of meals incorporating senior-friendly foods. In Korea, senior-friendly foods refer to products developed to facilitate chewing, swallowing, and digestion among older adults. These products are designated under the Aging-Friendly Industry Promotion Act and are intended to enhance safety and convenience in meal consumption. To minimize confounding due to changes in overall menu composition, senior-friendly items replaced comparable dishes within the existing meal structure rather than altering the broader nutritional framework. However, these foods were specifically modified to facilitate chewing and swallowing, which may have improved meal accessibility and reduced eating-related burden among older adults. In addition, the intervention meals were designed to maintain overall nutritional and caloric equivalence to the standard meals while providing adequate protein content to support muscle maintenance. Nutritional composition of the meals was evaluated in advance based on menu and product information. Dietary adherence was monitored through regular attendance at the welfare center and participation in the provided meals during the intervention period.

A one-week washout period was implemented between phases. Although this period appears short relative to conventional crossover trials, dietary interventions do not involve persistent biological residuals, and the washout period was considered sufficient to remove any residual effects of prior dietary exposure.

### 3.2 Data and measures

The primary outcome variables in this study are utility weight and quality-adjusted life expectancy (QALE). Utility weight captures health-related quality of life at a given point in time, while QALE translates this information into a measure that incorporates remaining life expectancy.

Utility weight was measured using the EQ-5D-5L instrument. The EQ-5D was developed by the EuroQol Group to assess health states across five dimensions: mobility, self-care, usual activities, pain/discomfort, and anxiety/depression. Each dimension consists of five levels of severity: no problems, slight problems, moderate problems, severe problems, and extreme problems. Participants reported their health status by selecting the level that best described their condition in each dimension.

After collecting responses, utility weights were calculated using the Korean EQ-5D-5L valuation set derived from a representative sample using the time trade-off (TTO) method. The computation was performed using the official valuation algorithm implemented in the corresponding application. The resulting utility weight ranges from values below zero (health states considered worse than death) to one (full health).

To extend the analysis beyond point-in-time health status, we constructed quality-adjusted life expectancy (QALE). QALE combines the estimated utility weight with remaining life expectancy, thereby translating contemporaneous changes in health-related quality of life into quality-adjusted life years. Remaining life expectancy was obtained from the 2022 Korean life tables published by Statistics Korea (25), matched to each participant by age and sex (25).

Let $W_{U}$ denote the individual’s utility weight and LY denote remaining life expectancy. In the simplest formulation without discounting, QALE can be expressed as:


$$\mathrm{QAL}E_{\mathrm{LY}}=W_{U}\times \mathrm{LY}$$

Because QALE represents the present value of future health, future health states are typically discounted. Following Sassi (30), discounted QALE can be expressed as:


$$\text{QALE}=Q\frac{1-e^{-\mathrm{rL}}}{r}$$

where Q denotes the utility weight, L denotes remaining life expectancy, and r is the discount rate. In this study, a discount rate of 3 percent was applied, consistent with commonly accepted practice in health economic evaluation (14, 26).

To quantify the effect of the senior-friendly diet on QALE, we use the change in utility weight estimated from the FE models. Let ΔQ denote the estimated increase in utility weight associated with exposure. Assuming that the intervention does not directly alter remaining life expectancy, the corresponding change in discounted QALE is given by:


$$\text{ΔQALE}=\mathrm{\Delta Q}\frac{1-e^{-\mathrm{rL}}}{r}$$

**Figure 2:**
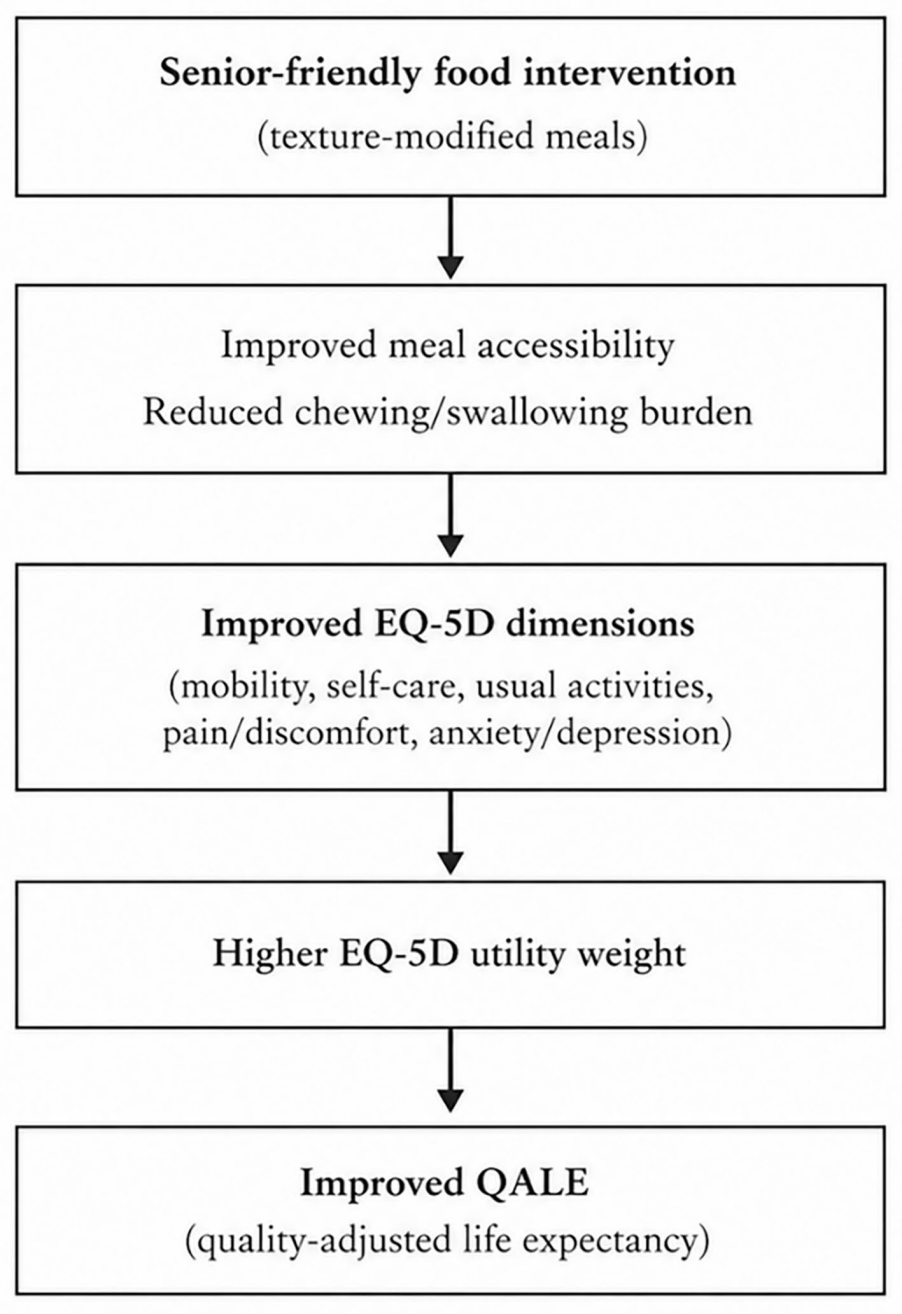
Conceptual framework linking the senior-friendly food intervention to QALE outcomes.

While dietary improvements can influence longevity for the long time, the 10-week intervention period in this study is unlikely to produce significant changes in life expectancy. Under this formulation, improvements in utility weight during the exposure period translate proportionally into gains in discounted quality-adjusted life expectancy (see Figure 2).

The primary explanatory variable captures exposure to the senior-friendly diet. In the difference-in-differences specification, we included indicators for treatment assignment, post-intervention period, and their interaction. In the fixed-effects specification, we included a contemporaneous exposure indicator and, in extended models, a carryover term to account for potential residual effects across periods.

We controlled individual characteristics that may influence health-related quality of life, including gender, age group, marital status, living arrangement (living alone or with others), income level, and education level. In addition, we included a measure of health status, specifically chewing ability, to account for functional oral health constraints that were not directly determined by random assignment but are plausibly related to both diet consumption and quality-of-life outcomes.

Although chewing ability was measured at each wave, it showed no within variation across the three waves. This result confirms that it is a time-invariant baseline characteristic rather than a post-treatment outcome. Thus, its inclusion as a covariate does not generate post-treatment bias into the estimates.

### 3.3 Econometric model

To evaluate the utility weight and benefits derived from the consumption of senior-friendly foods, we performed random assignments so that every participant has an equal chance of being in either the experimental or control group. However, there would be uncertainty in controlling observed and unobserved factors because the assignment was not performed under laboratory conditions and carryover effects may exist. Given the crossover structure of the study and the observed baseline imbalance in outcome measures, individual fixed-effects (hereafter FE) models serve as the primary analytical approach. FE models exploit within-person variation across all measurement occasions and remove time-invariant individual heterogeneity whether observed or unobserved, making them directly suited to the crossover design. To provide robustness evidence and interpretable time-window contrasts, OLS difference-in-differences (hereafter DID) models are estimated as a secondary analysis. Both approaches identify treatment effects from within-person or within-group changes over time rather than cross-sectional level differences, making them robust to the observed baseline imbalance in outcome measures.

The primary analysis employs individual fixed-effects models that make use of all three measurement occasions. These models focus on within-person changes and remove all time-invariant individual characteristics, whether observed or unobserved. This is particularly relevant in a crossover setting, where the same participant is observed under different exposure conditions across phases. The baseline fixed-effects model is as follows:


$$Y_{\mathrm{it}}=\alpha_{i}+\text{δExposur}e_{\mathrm{it}}+\lambda_{t}+\varepsilon_{\mathrm{it}},$$

where $\alpha_{i}$ denotes individual fixed effects and $\lambda_{t}$ denotes period fixed effects. The indicator $\text{Exposur}e_{\mathrm{it}}$ equals one when participant i consumes the senior-friendly diet at time t . The coefficient δ captures the contemporaneous association between exposure and the outcome after controlling for common time effects. In this specification, identification comes from participants switching exposure status across phases, which is a key feature of the crossover design.

Although the study included a washing-out period, residual effects from prior exposure remain possible. To assess whether such effects influence the estimates, we estimate an extended fixed-effects model that adds a carryover term:


$$Y_{\mathrm{it}}=\alpha_{i}+\text{δExposur}e_{\mathrm{it}}+\text{θCarryover}+\lambda_{t}+\varepsilon_{\mathrm{it}},$$

where $\text{Carryove}r_{\mathrm{it}}$ equals one if the participant consumed the senior-friendly diet in the previous phase. The coefficient θ captures potential lagged effects after exposure ends, while δ continues to represent the contemporaneous effect during exposure. This specification is intended to separate the immediate association of current exposure from any residual influence of prior exposure, recognizing that the two are mechanically related in a short panel with crossover assignment. This also serves as an empirical test of whether the one-week washout period was sufficient to remove residual effects of prior exposure.

As the secondary robustness analysis, we estimate a difference-in-differences (DID) framework using ordinary least squares. Let $Y_{\mathrm{it}}$ denote the outcome for individual i measured at time t . Outcomes include EQ-5D–based utility weight, QALE, and the five EQ-5D dimension scores. The DID approach compares changes in outcomes over time between participants assigned to the senior-friendly diet and those assigned to the general diet with parallel trends assumption. The baseline DID specification is as follows:


$$Y_{\mathrm{it}}=\alpha +\beta_{1}\text{Trea}t_{i}+\beta_{2}\mathrm{Pos}t_{t}+\beta_{3}(\text{Trea}t_{i}\times \mathrm{Pos}t_{t})+X_{i}^{\prime }\gamma +\varepsilon_{\mathrm{it}}$$

In this model, ${\text{Treat}}_{i}$ indicates assignment to the senior-friendly diet in the relevant phase, and ${\text{Post}}_{t}$ indicates the post-intervention measurement within a given comparison window. The interaction term $\text{Trea}t_{i}\times \mathrm{Pos}t_{t}$ captures the DID estimator, and the coefficient $\beta_{3}$ is interpreted as the average difference in outcome changes between treated and control participants over the same window. The vector $X_{i}$ includes observed individual characteristics, including gender, age group, marital status, living arrangement, income, education level, and chewing ability. These covariates are included to improve precision and to account for observable differences that may remain after random assignment in a modest sample. Standard errors are clustered at the individual level to account for repeated observations within individuals.

The validity of the DID model relies on the parallel trends assumption, which requires that outcomes would have evolved similarly across groups in the absence of treatment. Because the experiment follows a crossover structure, a single DID estimate is insufficient to summarize exposure over time. We therefore estimate separate DID models for two time windows: an initial effect comparing the first and second survey, capturing short-term changes during the first exposure period, and a cumulative effect comparing the first and third surveys. Because both groups receive the senior-friendly diet at different points in time, partial convergence in outcomes is expected by the end of the study, and a smaller cumulative estimate relative to the initial estimate is consistent with the crossover structure rather than a reversal of the effect.

In addition to the composite outcomes, the same FE and DID models are applied to each of the five EQ-5D dimensions separately to examine which specific domains drive the overall improvements in utility weight. Because higher scores in each dimension indicate more severe problems, negative coefficients imply improvements in health status.

Taken together, the fixed-effects models serve as the primary specification, exploiting within-person variation across all measurement occasions and controlling for time-invariant individual heterogeneity. The OLS-DID estimates across time windows provide complementary robustness evidence through interpretable contrasts tied to the timing of exposure and demonstrate robustness to the observed baseline imbalance in outcome measures.

To assess the sensitivity of the QALE estimates to the choice of discount rate, we re-estimate QALE using discount rates of 0 and 5% in addition to the baseline rate of 3%. Under a 0% discount rate, QALE simplifies to the product of utility weight and remaining life expectancy. Under a 5% discount rate, future health gains are discounted more heavily. Comparing results across the three discount rates allows us to evaluate whether the main findings are robust to this assumption.

Since the sample size was determined by participant availability rather than a formal *a priori* power calculation, a *post hoc* power analysis was conducted to retrospectively assess the adequacy of the sample. Achieved power was computed from the observed t-statistic, defined as the ratio of the estimated coefficient to its standard error from the primary fixed-effects estimates, under a two-sided test at the 5% significance level.

### 3.4 Ethical considerations

This study was approved by the Institutional Review Board of Kyung Hee University (KHGIRB-23-276). Written informed consent was obtained from all participants prior to participation. All procedures involving human participants were conducted in accordance with the ethical standards of the institutional research committee and the Declaration of Helsinki.

## 4 Results

### 4.1 Descriptive statistics

Table 1 presents descriptive statistics by group at baseline. The two groups are well balanced in observable individual characteristics. For example, the proportion of females is similar across groups (0.518 in Group 1 vs. 0.491 in Group 2, *p* = 0.774), as is mean age (74.0 vs. 75.2 years, *p* = 0.270). No statistically significant differences are observed for marital status, living arrangement, income level, or education (Table 1).

**Table 1:** Descriptive statistics by group.

Variable	Group 1	Group 2	Difference	*p*-value
Individual characteristics				
Female (male)	0.518	0.491	−0.027	0.774
Age	74.0	75.2	1.200	0.270
Married (others)	0.667	0.684	0.018	0.844
Living with partners (without)	0.352	0.298	−0.054	0.547
Low income (high)	0.370	0.404	0.033	0.720
High school or above (below)	0.463	0.491	0.028	0.766
Individual health				
Ability to chew (inability)	0.719	0.759	−0.040	0.632
Quality of live				
Utility weights	0.951	0.906	−0.045*	0.049
QALE	13.6	11.8	−1.7†	0.085

**p < 0.01; *p < 0.05; ^†^*p* < 0.1.

However, baseline differences are observed in the outcome measures. Mean utility weight is higher in Group 1 than in Group 2 (0.951 vs. 0.906, *p* = 0.049), and QALE is also higher in Group 1 (13.6 vs. 11.8), with the difference being marginally significant (*p* = 0.085). These baseline differences motivate the use of difference-based estimators in the subsequent analysis.

### 4.2 FE estimates

The primary analysis estimates 3-period individual fixed-effects models (time = 0, 1, 2). In these models, treatment indicates contemporaneous exposure to the senior-friendly diet, time indicators capture common period effects, and the carryover indicates exposure in the previous period, shown in Table 2.

**Table 2:** Estimation result of FE in utility weight and QALE.

Variable	Without carryover		With carryover	
	Utility weight	QALE	Utility weight	QALE
Experiment status				
Treated (untreated)	0.053** (0.013)	0.655** (0.142)	0.055** (0.021)	0.731** (0.231)
Period 2 (period 1)	0.026† (0.014)	0.220 (0.152)	0.025 (0.016)	0.184 (0.178)
Period 3 (period 1)	0.010 (0.012)	0.031 (0.123)	0.007 (0.029)	−0.081 (0.315)
Carryover	–	–	0.004 (0.037)	0.151 (0.388)

**p < 0.01; *p < 0.05; ^†^*p* < 0.1.

Period 1 represents the experiments performed before wahsingout while period 2 after washingout.

Without including the carryover term, the estimated contemporaneous treatment effect is positive and statistically significant for both outcomes. The coefficient on treatment effect is 0.053 (SE = 0.013, *p* < 0.01) for utility weight and 0.655 (SE = 0.142, *p* < 0.01) for QALE. When the carryover term is included, the estimated contemporaneous effect remains positive and statistically significant, with coefficients of 0.055 (SE = 0.021, *p* < 0.01) for utility weight and 0.731 (SE = 0.231, *p* < 0.01) for QALE. The similarity of the treatment estimates across specifications suggests that the estimated contemporaneous effect is robust to whether lagged exposure is modeled explicitly.

The carryover coefficient itself is small and not statistically significant in either outcome (0.004, SE = 0.037 for utility weight; 0.151, SE = 0.388 for QALE). This provides limited evidence that prior exposure generates additional residual improvements beyond the contemporaneous exposure period, although the precision of this estimate is constrained by the crossover structure and the resulting correlation between current exposure and lagged exposure. The time indicators are generally small and not statistically significant, indicating that common period effects do not account for the main changes in the outcomes once contemporaneous exposure is included.

Taken together, the fixed-effects estimates indicate that exposure to the senior-friendly diet is consistently associated with statistically significant improvements in utility-based quality of life and QALE.

The statistically significant results across both primary outcomes of utility weight and QALE indicate that the sample was sufficient to detect the observed treatment effects. *Post hoc* power analysis confirms this, with achieved power of 74.5% for utility weight and 88.6% for QALE at the 5% significance level.

Also, the carryover coefficient was small and statistically non-significant for both utility weight and QALE, providing empirical evidence supporting the adequacy of the washout period.”

### 4.3 OLS-DID estimates

As the secondary robustness analysis, Table 3 reports OLS-DID estimates for two-time windows using EQ-5D-DL outcomes. The first window compares the first and second survey and is interpreted as the initial effect of the senior-friendly diet. The second window compares the first and third survey and is interpreted as the cumulative effect over the full study period (Table 3).

**Table 3:** Estimation result of OLS-DID in utility weight and QALE.

Variable	Initial effect		Cumulative effect	
	Utility weight	QALE	Utility weight	QALE
Experiment status				
Treated (untreated)	−0.013 (0.022)	0.452 (0.659)	0.012 (0.022)	−0.465 (0.659)
After treatment (before)	0.025 (0.016)	0.184 (0.179)	0.011 (0.016)	0.070 (0.158)
Treatment × Time	0.055** (0.021)	0.731** (0.232)	0.051** (0.024)	0.580** (0.250)
Individual health				
Ability to chew (inability)	0.070** (0.031)	1.631* (0.908)	0.043** (0.019)	1.309 (0.883)

**p < 0.01; *p < 0.05; ^†^*p* < 0.1.

Individual characteristics and chewing ability were included as covariates. Among these variables, gender, age group, marital status, cohabitation status, and income level were not statistically significant and are therefore omitted from the table.

In the initial window (the first - second survey), the treatment–time interaction is positive and statistically significant for both outcomes. The estimated coefficient is 0.055 (SE = 0.021, *p* < 0.01) for utility weight and 0.731 (SE = 0.232, *p* < 0.01) for QALE. These estimates indicate that, over the initial exposure period, the treated group experienced larger improvements than the untreated group. The coefficients on the treatment indicator and the post-period indicator are not statistically significant, suggesting no meaningful baseline differences between groups and no strong common time trend in this window.

In the cumulative window (the first – third survey), the treatment–time interaction remains positive and statistically significant. The estimated coefficient is 0.051 (SE = 0.024, *p* < 0.05) for utility weight and 0.580 (SE = 0.250, *p* < 0.05) for QALE. The direction of the effects is the same as in the initial window, while the magnitude is somewhat smaller. This attenuation is consistent with the crossover structure, in which both groups are exposed to the senior-friendly diet at different points in time, leading to partial convergence in outcomes over the full period. Overall, the OLS-DID results indicate statistically significant improvements following initial exposure and positive cumulative differences when comparing the beginning and the end of the study.

Chewing ability is strongly associated with both outcomes in both specifications. The coefficient is positive and statistically significant for utility weight (0.070, SE = 0.031, *p* < 0.05 in the initial window; 0.043, SE = 0.019, *p* < 0.05 in the cumulative window). The corresponding estimates for QALE are also positive, although not precisely estimated. These results are consistent with chewing-related functional status being an important determinant of quality-of-life measures and justify its inclusion as a covariate.

The OLS-DID results are consistent with the FE estimates, confirming statistically significant improvements in utility weight and QALE and supporting the robustness of the primary findings.

### 4.4 EQ-5D dimension

**Figure 3:**
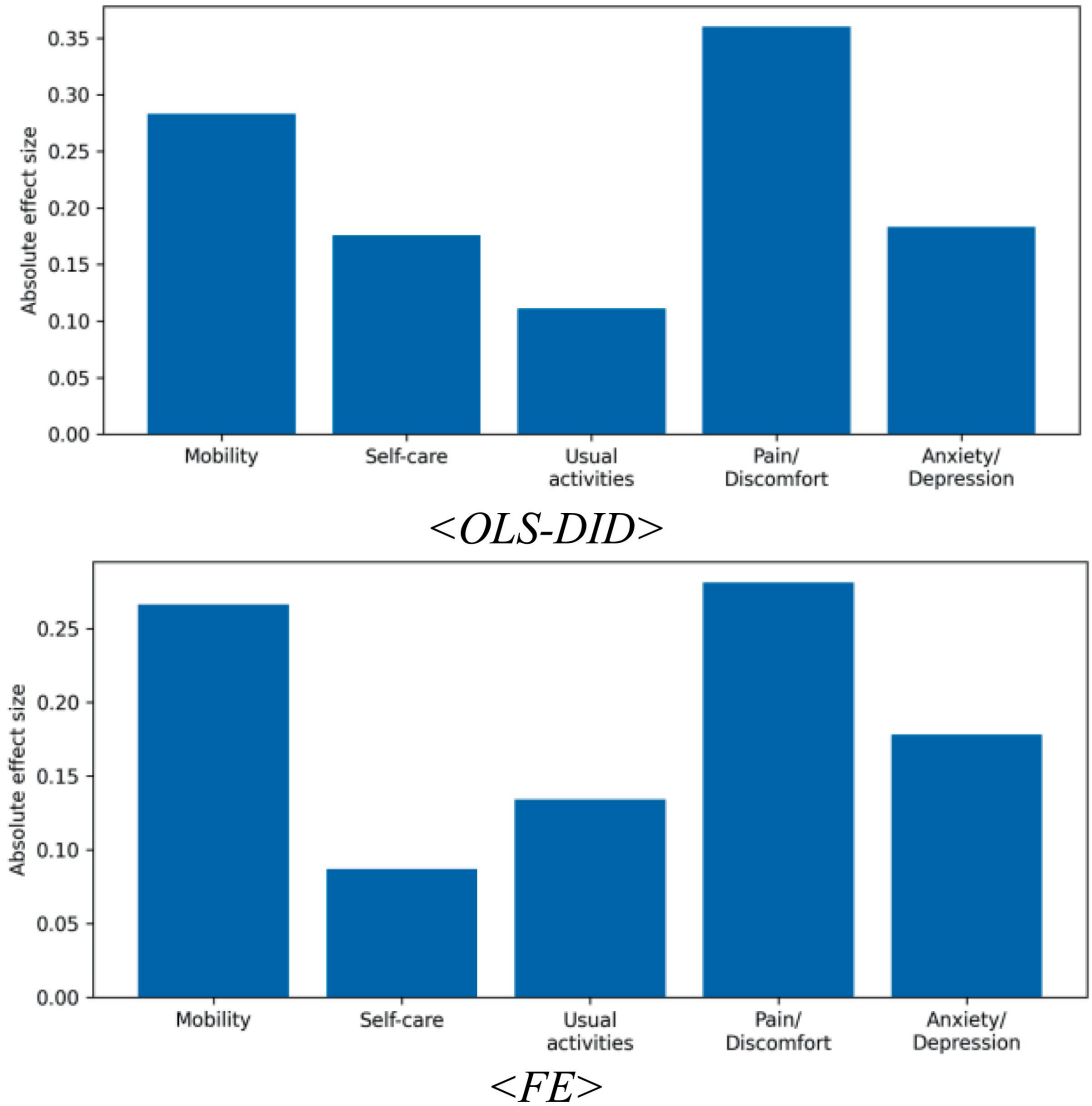
Estimation result in EQ-5D-DL subsection.

Tables 4, 5 report dimension-specific estimates for the EQ-5D-5L outcomes using the FE and OLS-DID models, respectively. Because higher scores in each EQ-5D dimension indicate more severe problems, negative coefficients imply improvements (see Figure 3).

**Table 4:** Estimation result of FE in EQ-5D-DL subsection.

Variable	Mobility	Self-care	Usual activities	Pain/discomfort	Anxiety/depression
Experiment status					
Treated (untreated)	−0.266** (0.070)	−0.087* (0.038)	−0.134** (0.043)	−0.281** (0.077)	−0.178** (0.055)
2nd period (1st period)	0.202* (0.087)	0.115** (0.050)	0.011 (0.054)	0.155 (0.100)	0.035 (0.065)
3rd period (1st period)	0.047 (0.079)	0.009 (0.027)	−0.049 (0.061)	−0.126 (0.090)	−0.053 (0.061)

**p < 0.01; *p < 0.05; ^†^*p* < 0.1.

**Table 5:** Estimation result of OLS-DID in EQ-5D-DL subsection.

Variable	Mobility	Self-care	Usual activities	Pain/discomfort	Anxiety/depression
Experiment status					
Treated (untreated)	0.083 (0.122)	0.005 (0.044)	0.017 (0.096)	0.078 (0.145)	0.008 (0.122)
After treatment (before)	0.211† (0.115)	0.158* (0.061)	0.000 (0.067)	0.193 (0.129)	0.035 (0.071)
Treatment × Time	−0.285* (0.139)	−0.176** (0.063)	−0.111 (0.092)	−0.360* (0.169)	−0.183 (0.114)
Individual health					
Inability to chew	−0.291† (0.156)	−0.085 (0.078)	−0.163 (0.099)	−0.463** (0.165)	−0.236 (0.146)

**p < 0.01; *p < 0.05; ^†^*p* < 0.1.

Table 4 presents the results from the 3-period individual fixed-effects models, which exploit within-individual variation over time and control for time-invariant unobserved heterogeneity. The treatment indicator is negative across all five EQ-5D dimensions and statistically significant at conventional levels. The estimated effects are −0.266 (*p* < 0.05) for mobility, −0.087 (*p* < 0.05) for self-care, −0.134 (*p* < 0.05) for usual activities, −0.281 (*p* < 0.05) for pain/discomfort, and −0.178 (*p* < 0.05) for anxiety/depression.

In the OLS-DID specification (Table 5), the interaction term between treatment and time is negative and statistically significant in several physical dimensions. Specifically, statistically significant reductions are observed in mobility (−0.283, *p* < 0.05), self-care (−0.176, *p* < 0.01), and pain/discomfort (−0.360, *p* < 0.05). The estimate for usual activities (−0.111) is marginally significant at the 10 percent level. In contrast, the coefficient for anxiety/depression (−0.183) is not statistically significant. The direction of the effects is consistent with the FE estimates, while statistical significance is somewhat less uniform across dimensions in the OLS-DID specification.

Overall, the dimension-specific results indicate that exposure to the senior-friendly diet is associated with reductions in reported problems across multiple domains of health-related quality of life. The pattern is statistically significant across all five dimensions in the FE models, and is most pronounced in physical functioning and pain-related dimensions in the OLS-DID models.

Table 6 reports QALE estimates and the corresponding treatment effects under discount rates of 0, 3, and 5%. The estimated treatment effects remain positive and statistically significant across all three specifications. The magnitude of the effect decreases as the discount rate increases, which is expected given that higher discount rates reduce the present value of future health gains. These results indicate that the main findings are not sensitive to the choice of discount rate.

**Table 6:** Sensitivity analysis for QALE: FE estimates of QALE under alternative discount rates.

Variable	Without carryover			With carryover		
	0% discount	3% discount	5% discount	0% discount	3% discount	5% discount
Without carryover						
Treated (untreated)	0.831** (0.176)	0.655** (0.142)	0.567** (0.124)	0.944** (0.292)	0.731** (0.231)	0.625** (0.200)
Period 2 (period 1)	0.268 (0.189)	0.220 (0.152)	0.196 (0.133)	0.212 (0.223)	0.184 (0.178)	0.168 (0.156)
Period 3 (period 1)	0.023 (0.155)	0.031 (0.123)	0.034 (0.108)	−0.146 (0.397)	−0.081 (0.315)	−0.052 (0.275)
Carryover	–	–	–	0.227 (0.486)	0.151 (0.388)	0.117 (0.338)

**p < 0.01; *p < 0.05; ^†^*p* < 0.1.

Period 1 represents the experiments performed before wahsingout while period 2 after washingout.

## 5 Discussion

This study evaluates whether a senior-friendly diet is associated with improvements in EQ-5D-DL outcomes under a crossover structure. The main empirical finding is consistent across specifications. The senior-friendly diet is associated with positive and statistically significant improvements in both utility weight and QALE.

The fixed-effects results provide the primary evidence for this conclusion by relying on within-person changes and by using all three measurement occasions. The contemporaneous exposure indicator is robustly associated with higher utility weight and higher QALE. Importantly, this result holds whether a carryover term is included. This robustness reduces concerns that the estimated association is an artifact of a particular time-window choice or baseline differences across groups. The carryover coefficient is not statistically significant, which suggests limited evidence of residual gains persisting beyond the exposure period after controlling contemporaneous exposure. At the same time, this result should not be interpreted as evidence that carryover is absent. In a crossover setting with a small number of periods, current exposure and lagged exposure are mechanically related, which can reduce the precision of the carryover estimate. Therefore, the main conclusion supported by the data is about contemporaneous improvements during exposure, rather than long-lasting residual effects after exposure.

The OLS-DID results provide clear time-window interpretation. In the initial comparison between the first and second survey, the treated group shows larger improvements than the untreated group, indicating that measurable benefits can emerge during the first exposure period. In the cumulative comparison between the first and third survey, the estimated effects remain positive and statistically significant, indicating that the overall differences between the beginning and end of the study are in the same direction. The smaller magnitude in the cumulative window is expected under a crossover design. Because both groups are exposed to the senior-friendly diet at different points in time, outcomes may partially converge over the full period. Under this interpretation, attenuation in the first-third survey estimates reflect partial equalization rather than a reversal of the effect.

From a substantive perspective, the consistency across utility weight and QALE is informative. Utility weight captures changes in health-related quality of life, while QALE translates these changes into a measure that incorporates remaining life expectancy. The use of QALYs in older populations has been subject to ongoing debate. Previous studies have suggested that QALY-based evaluations may undervalue health gains in older adults due to lower baseline utility scores and potential age-related bias in preference weights (29). Despite these concerns, QALY remains a widely accepted outcome measure in health economic evaluations, allowing comparability across studies and interventions. In this study, QALY was used primarily to assess within-group changes over time rather than absolute comparisons between age groups, which may mitigate some of the inherent limitations associated with baseline utility differences. In this context, the consistency between utility weight and QALE further strengthens the interpretation of the findings. The fact that both outcomes move in the same direction suggests that the senior-friendly diet is associated with improvements that are relevant both in terms of current quality of life and in terms of quality-adjusted life expectancy. However, the estimated QALE gains should be interpreted cautiously because QALE projections inherently rely on assumptions regarding the persistence and duration of observed utility changes over time. Therefore, the findings are better interpreted as projected associations under the observed study conditions rather than definitive long-term causal effects. The covariate results further support the plausibility of the findings. Chewing ability is strongly related to utility weight, indicating that functional oral health constraints are closely linked to quality-of-life outcomes in this sample and that controlling for this factor is important.

The sensitivity analysis across discount rates of 0, 3, and 5% confirms that the QALE-based findings are robust to this assumption. Although the magnitude of the estimated effect varies with the discount rate, the direction and statistical significance remain consistent across specifications.

Several design features should be kept in view when interpreting the results. First, the study follows a crossover structure, and both groups receive the senior-friendly diet at some point. For this reason, comparisons over the full period are best interpreted as reflecting differences in cumulative exposure and exposure timing rather than clean period-specific causal effects. Second, although a washout period was implemented, outcomes were not measured immediately after washout, limiting the ability to isolate post-washout baselines. This motivates the focus on the first-second and first-third survey comparisons and the use of fixed-effects models that explicitly separate contemporaneous exposure from lagged exposure. Third, the analysis is based on three measurement occasions, which restrict the ability to model richer dynamics and may limit power for detecting carryover effects. In addition, the relatively short follow-up period limits the ability to determine whether the observed improvements in utility weight and QALE would persist over longer time horizons.

Despite these limitations, the empirical pattern is consistent. The senior-friendly diet is associated with improvements during the exposure period, and the cumulative comparison indicates that differences between the beginning and end of the study remain positive even under crossover exposure. These findings suggest the potential practical relevance of senior-friendly dietary interventions in relation to health-related quality of life among older adults, while also indicating that future studies with more frequent measurement around washout and longer follow-up would be useful for clarifying the persistence of effects after exposure ends.

The fixed-effects results reinforce this interpretation while providing a broader pattern of statistical significance. When individual fixed effects are introduced and the analysis relies on within-person variation across all three periods, the treatment indicator is negative and statistically significant across all five EQ-5D dimensions. The direction of the effects is consistent with the OLS-DID results, and the magnitude of the coefficients is comparable across specifications. The fact that statistical significance becomes more uniform in the fixed-effects model suggests that baseline heterogeneity across individuals may attenuate the precision of dimension-specific estimates in the simpler difference-in-differences framework. By controlling time-invariant individual characteristics, the fixed-effects model isolates the contemporaneous association between exposure and within-person changes in each health dimension.

In addition to the composite measures, the dimension-specific results provide further insight into the mechanism through which the senior-friendly diet is associated with improvements in health-related quality of life. The OLS-DID estimates indicate that the effects are concentrated primarily in the physical dimensions of the EQ-5D. Mobility, self-care, and pain/discomfort show statistically significant reductions in reported problems, and usual activities show a marginally significant reduction. In contrast, anxiety/depression does not show a statistically significant effect in the OLS-DID specification. This pattern suggests that the observed improvements in utility weight are driven largely by changes in physical functioning and pain-related domains rather than mental health dimensions.

Taken together, the dimension-level findings clarify the structure underlying the improvements observed in utility weight and QALE. Utility weight is a weighted aggregation of the five EQ-5D dimensions, and QALE translates this composite index into a quality-adjusted life expectancy measure. The concentration of statistically significant effects in mobility, self-care, and pain/discomfort indicates that the gains in composite outcomes are rooted in functional and pain-related improvements. These domains are directly linked to nutritional intake, physical strength, and daily functioning in older adults. The relative weakness of the anxiety/depression effect in the OLS-DID specification is consistent with the idea that short-term dietary interventions may influence physical functioning more directly than psychological states. The fixed-effects results suggest that even the mental health dimension may improve during exposure, but the evidence is less robust than for physical domains.

Overall, the composite and dimension-specific analyses present a coherent pattern. Improvements in mobility, self-care, usual activities, and pain/discomfort provide the micro-level basis for the observed increases in utility weight and QALE. The findings are internally consistent across modeling strategies and support the interpretation that the senior-friendly diet may contribute to improvements in health-related quality of life during the exposure period.

## 6 Conclusion

This study examined the association between a senior-friendly diet and health-related quality of life among older adults receiving community-based care. Exposure to the senior-friendly diet was associated with statistically significant increases in utility weight and QALE. The dimension-specific analysis indicates that these improvements were observed mainly in mobility, self-care, usual activities, and pain/discomfort, while the evidence for anxiety/depression is weaker. These results suggest that the observed gains in the composite measures are largely driven by improvements in physical functioning and discomfort-related components.

Since the study follows a crossover design, both groups received the intervention at different points in time. Accordingly, the estimates are interpreted in terms of exposure timing and effects during the exposure period rather than permanent differences between groups. The initial time-window comparison captures short-term improvements during the first exposure period, whereas the cumulative comparison reflects differences in exposure timing over the full study period. The fixed-effects models, which rely on within-person variation, yield results consistent with the DID estimates and provide limited evidence of carryover effects after exposure ends.

These findings are relevant to community-based care settings for older adults. Improvements in mobility, self-care, usual activities, and pain/discomfort are closely related to the level of assistance older adults require in daily life and may help them maintain functional independence. Although this study does not estimate cost implications directly, the increase in QALE provides a quantitative measure linking dietary exposure to quality-adjusted health outcomes in a welfare center setting.

Several limitations should be noted. First, the crossover design does not allow for a permanently untreated control group, and the estimates rely on variation in exposure timing. Second, the OLS-DID robustness analysis depends on the parallel trends assumption, which cannot be verified directly in a shared community environment. Third, outcomes were not measured immediately after the washing-out period, which limits the ability to isolate post-washout baselines and assess longer-term persistence. Fourth, the analyses across five EQ-5D dimensions involve multiple comparisons, raising the risk of false positives. As these analyses are exploratory rather than hypothesis, results should be interpreted with caution. Finally, this study focused on self-reported EQ-5D outcomes. Although precise individual-level meal intake records were not systematically collected, all meals were provided directly through the welfare center meal service system, and participants attended the center regularly during the intervention period. Therefore, some caution is warranted when interpreting the findings in relation to individual-level dietary adherence. In addition, the 10-week intervention period may not have been sufficient to produce significant changes in objective health indicators. While objective measures such as biomarkers or physical performance tests were not used, the 10-week intervention period may not be sufficient to produce significant changes in such indicators. Future studies incorporating objective health measures would help clarify the pathways through which senior-friendly diets influence health-related quality of life.

Despite these limitations, the empirical results are consistent across model specifications. The senior-friendly diet is associated with significant improvements in health-related quality of life during the exposure period. Further research with longer follow-up periods and explicit cost-effectiveness analysis would help clarify the persistence and economic relevance of these effects.

## Data Availability

The data supporting the findings of this study are not publicly available due to privacy and ethical restrictions. Requests for access to the data should be directed to the corresponding author and may require approval from the relevant research funding or supporting organizations, as well as compliance with applicable ethical requirements.
